# Dhoti cancer: a waistline skin cancer with review of literature

**DOI:** 10.1186/s12957-015-0698-z

**Published:** 2015-09-22

**Authors:** Murtaza A. Akhtar, Divish K. Saxena, Akanksha A. Chikhlikar, Akshay P. Bangde, Murtuza Rangwala

**Affiliations:** Department of Surgery, NKP Salve Institute of Medical Sciences and Research Centre, Lata Mangeshkar Hospital, Digdoh hills, Hingna Road, Nagpur, 440019 India

**Keywords:** Waistline cancer, Dhoti cancer, Squamous cell carcinoma skin, Saree cancer

## Abstract

Skin cancers account for less than 1 % of all malignancies in India. Squamous cell carcinomas occurring over the waistline due to tying of cotton cloth called dhoti in males and sarees in females are predominantly seen in traditional Indian population. On wearing of these clothes for years, there is a constant irritation which produces depigmentation, glazing of the skin, acanthosis, scar formation, and later on malignant transformation. Presenting a case of a 65-year-old male with 7 × 5 cm ulceroproliferative growth over the right waistline with a history of prolonged use of dhoti. Wide local excision of the growth with 2-cm margin and primary closure of wound by mobilizing the skin was carried out. Histopathology showed well-differentiated squamous cell carcinoma. The patient is clinically disease free after postoperative follow-up of 1 year.

## Background

Waistline cancer is a rare type of squamous cell cancer occurring over the waistline predominantly in Indian males and females wearing a piece of cotton cloth used to cover the lower part of the body. This cloth is called dhoti in men and saree in women. The dhoti is worn tightly around the waist with one of the shorter ends carried under the groin and tucked at the back, and the saree is adorned with a petticoat worn underneath by women, which is secured tightly to the waist by a cord. The tight knot around the waist at a similar position causes long-term friction and along with perspiration leads to pigmentation and scale-like changes in the skin which eventually undergo malignant transformation. The knowledge and existence of such malignancy is sparse in Indian medical fraternity [1] where it is predominantly observed, and hence, there is a need to publish this case and review literature.

## Case presentation

**Fig. 1:**
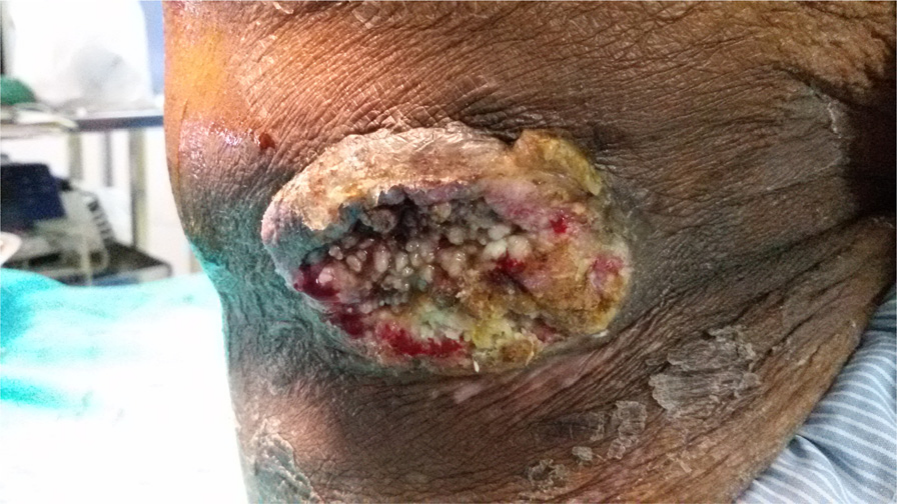
Photograph showing ulceroproliferative growth over the right waist with scaling of the skin

A 65-year-old male patient presented with a progressively increasing painless ulcer on the right side of the waist with a 6-month duration. Patient gave history of tightly tying a cotton dhoti over the waistline for the last 40 years. On local examination, there was a hypopigmented skin patch with scaling and ulceroproliferative growth of 7 × 5 cm with everted edges (Fig. 1). There was no significant inguinal lymphadenopathy noted clinically and confirmed by FNAC. Changes of hypopigmented skin without ulceration were also observed on the left waistline. Based on the clinical suspicion, patient underwent wide local excision with clearance margin of 2 cm and primary closure of skin defect by mobilizing the skin flap. As regional lymph nodes were clinically and cytologically negative, they were kept under clinical surveillance.

**Fig. 2:**
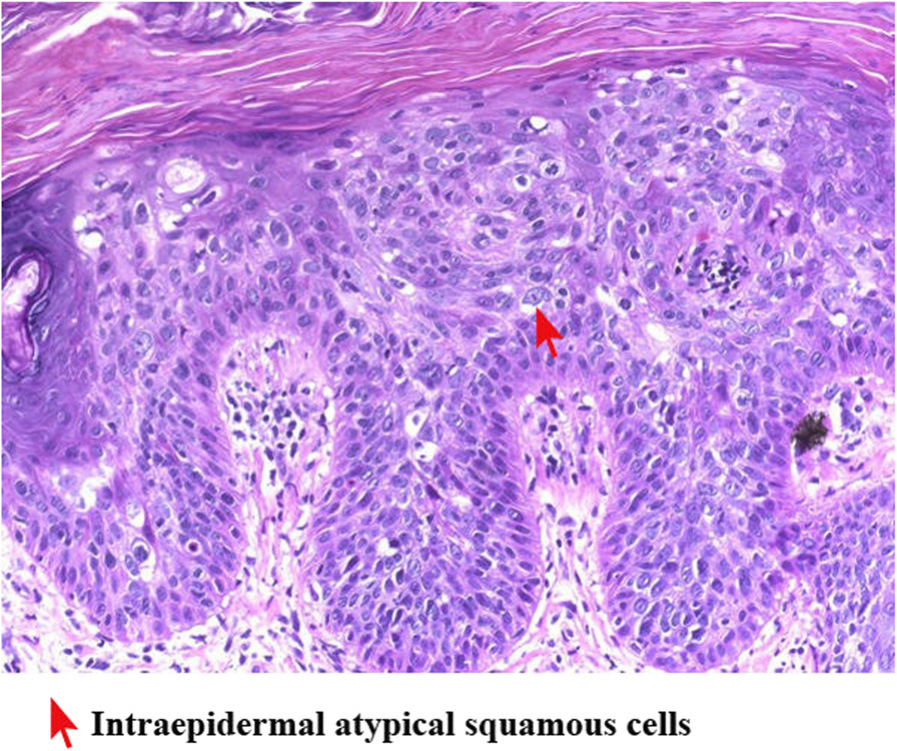
Photograph showing histopathological features of squamous cell carcinoma

Histopathology reports confirmed the presence of squamous cell carcinoma with tumor-free margin (Fig. 2). Postoperatively, the wound healed well without any complication, and the patient was discharged with a 3-month follow-up regime. After 1 year, locoregional recurrence was not observed on clinical evaluation.

### Discussion

The term dhoti cancer was coined in 1945 by Khanolkar and Suryabai [2] which is a form of a waistline squamous cell carcinoma associated with wearing of dhoti by traditional Indian males. Similarly, wearing of saree by Indian women causing squamous cell carcinoma of the waistline was termed as “saree cancer” [3]. The data on this poorly understood squamous cell carcinoma of the skin is sparse in literature and hence the need for reporting this case with literature review.

The exact causation of dhoti cancer is ill understood, and there are many hypothesis put forward to explain its probable mode of causation. Constant friction at the site of tying of dhoti or saree causes friction of the waistline which is associated with dermatoses [4]. Other hypothesis for development of this waistline skin malignancy is continuous irritation which increases potential of malignant degeneration [5] which could also be due to altered cycle of damage, irritation, and repair leading to malignant transformation [6].

Another hypothesis put forward is depressed immunological state produced by the surrounding scar tissue [7] and absence of lymphatic drainage from a scar which causes a significant delay in the host immunologic recognition and the antitumor immunologic response [8]. Squamous cell carcinoma developing in the scar was described by Marjolin in 1828 [9], and saree and dhoti cancer could be a variant of scar cancer as constant irritation by dhoti produces depigmentation, glazing of the skin, acanthosis, scar formation, and later on malignant transformation that occurs in the scar. The dermal changes lead to scarring, and development of malignancy in this area of friction with scarring is the reason to call it as Marjolin-like cancer. A genetic hypothesis implicating human leukocyte antigen (HLA) DRG and mutation in P53 or FAS genes is also proposed [10] for malignancy in the scar [11].

The age of presentation in the present case that was 65 years with more than 40 years of wearing dhoti is quite consistent with its occurrence in the age group after 50 years as reported in literature. This suggests a prolonged exposure of more than 40 years before malignant transformation [12]. The features of these skin cancers are similar to Marjolin’s ulcer, being slow growing and well differentiated and lymph node metastasis is rare and that too will occur if growth infiltrates the surrounding skin or underlying muscle when inguinal lymph nodes will get involved requiring wide excision of the skin with grafting and radiotherapy to inguinal lymph nodes along with chemotherapy [13]. The treatment of a well-localized dhoti cancer is wide local excision with excision of the skin with acanthosis-like changes and primary closure or split skin grafting to cover the raw area. Long-term survival is not documented in literature due to the rarity of the disease. Another important aspect is follow-up of other side lesion where a change of acanthosis was observed. There is a need for an awareness drive for medical health providers and common public regarding these malignancies and regular screening of the waist skin of chronic *dhoti* users for any malignant transformation.

## Conclusions

A rare case of squamous cell carcinoma due to chronic friction by wearing dhoti is presented with a treatment plan. It is expected that with change of lifestyle and lesser use of dhoti in new-generation Indians, the occurrence of *dhoti* cancer will become further rare, but clinicians in India need to remember this condition as dhoti will continue to be a dress of traditional Indian males. The presence of acanthotic lesion in the waistline should be viewed with suspicion, and a constant monitoring to the patient is recommended.

## Consent

The patient has given a written consent. Written and informed consent was obtained from the patient for the publication of case report and accompanying images. A copy of written consent obtained in vernacular language (Marathi) is available with us and is being held back to protect patient’s identity. It could be submitted with name blocked if the journal requires it.

## References

[1] Naveen N, Kumar MK, Babu RK, Dhanraj P (2014). A rare case of synchronous saree cancer. J Cutan Aesthet Surg.

[2] Khanolkar VR, Suryabai B (1945). Cancer in relation to usages: three new types in India. Arch. Path.

[3] Patil AS, Bakhshi GD, Puri YS, Gedham MC, Naik AV, Joshi RK (2005). Saree cancer. Bombay Hosp J.

[4] Eapen BR, Shabana S, Anandan S (2003). Waist dermatoses in Indian women wearing saree. Indian J Dermatol Venereol Leprol.

[5] Dm G, Cl K (1949). Marjolin’s ulcer; a preventable threat to function and life. Am J Surg.

[6] Copcu E, Aktas A, Siman N, Oztan Y (2003). Thirty-one cases of Marjolin’s ulcer. Clin Exp Dermatol.

[7] Castillo J, Goldsmith HS (1968). Burn scar carcinoma. CA Cancer J Clin.

[8] Fishman JR, Parker MG (1991). Malignancy and chronic wounds: Marjolin’s ulcer. J Burn Care Rehabil.

[9] Trent JT, Kirsner RS (2003). Wounds and malignancy. Adv Skin Wound Care.

[10] Harland DL, Robinson WA, Franklin WA (1997). Deletion of the p53 gene in a patient with aggressive burn scar carcinoma. J Trauma..

[11] Lee SH, Shin MS, Kim HS, Park WS, Kim SY, Jang JJ (2000). Somatic mutations of the Fas (Apo-1/CD95) gene in cutaneous squamous cell carcinoma arising from a burn scar. J Invest Dermatol..

[12] Jagadishwar Goud G (2014). Waist line skin cancer: a case report. JMSR.

[13] Takalkar UV, Asegaonkar SB (2014). Saree cancer in Indian woman treated successfully with multimodality management. Dermatol Reports.

